# Effect of the Resorcinol/Formaldehyde Ratio and the Temperature of the Resorcinol–Formaldehyde Gel Solidification on the Chemical Stability and Sorption Characteristics of Ion-Exchange Resins

**DOI:** 10.3390/gels7040239

**Published:** 2021-11-27

**Authors:** Eduard Tokar, Mikhail Tutov, Pavel Kozlov, Arseni Slobodyuk, Andrei Egorin

**Affiliations:** 1 Institute of Chemistry, Far Eastern Branch, Russian Academy of Sciences, 159, Prosp. 100-Letiya Vladivostoka, 690022 Vladivostok, Russia; d.edd@mail.ru (E.T.); thunderbird87@mail.ru (M.T.); ampy@ich.dvo.ru (A.S.); 2 FSUE “Mayak Production Association”, The Department of Chemistry and Chemical Technology, Prosp. Lenina 31, 456780 Ozersk, Russia; kozlov_pavel@inbox.ru

**Keywords:** resorcinol–formaldehyde resin, adsorption, cesium, liquid radioactive waste

## Abstract

A series of resorcinol–formaldehyde resins (RFR) samples for Cs-137 removal from liquid alkaline media have been synthesized. It has been demonstrated that the chemical stability as well as sorption characteristics are determined by the resorcinol/formaldehyde molar ratio and the solidification temperature. It has been also demonstrated that the sample synthesized at the resorcinol/formaldehyde molar ratio of 1.8/2.2 and solidified at 210 °C is characterized by the best sorption-selective characteristics and chemical stability. Under dynamic conditions, at feeding >1000 bed volumes of a model solution with pH > 13, the RFR 3-1 goes through six sorption cycles without noticeable changes in the sorption characteristics. The results are presented that demonstrate the possibility of RFR application in the decontamination of real LRW from Cs-137.

## 1. Introduction

The processing of liquid radioactive waste generated by nuclear plants is an urgent technical and environmental task. Heterogeneous radioactive waste streams with high mineralization and pH are particularly hazardous. These waste streams are normally processed with the removal of Cs-137 radionuclide, which contributes up to 95–99% of the total radioactivity.

These wastes were formed during the implementation of the Manhattan Project and are stored and processed at the Savannah River Site (Aiken, SC, USA) and the Hanford Site (Benton County, WA, USA). The chemical composition of the clarified part of heterogeneous radioactive waste is mainly represented by Na^+^ salts (more than 1 mol/L) in the form of nitrites and nitrates as well as OH^-^ (more than 0.5 mol/L) [1,2,3,4,5]. Radioactive wastes (RAW) with similar composition of a volume of about 15,000 m^3^ are located in closed storage tanks at the Federal State Unitary Enterprise “Mayak Production Association” (Chelyabinsk oblast’, Russia) [6,7]. The clarified part of this waste comprises highly mineralized solutions, the chemical composition of which mainly consists of Na^+^ salts (3–4.5 mol/L) in the form of nitrates, hydroxides, aluminates, and nitrites [8].

These liquid radioactive wastes (LRW) are usually disposed by immobilization in cement matrices, but, prior to this, it is required to remove Cs-137 radionuclide, whose approximate activity is (3–5) × 10^10^ Bq/L. One should mention that to make the cement compound comply with the requirements for classification to Class 3 of RAW (according to the Decree of the Government of the Russian Federation No. 1069 of 19.10.2012), the activity of Cs-137 in the cement compound must be not higher than 1.0 × 10^10^ Bq/kg. Taking into account the dilution of LRW by the matrix components, the bulk activity of Cs-137 in the solidified cemented LRW must not exceed 3.0 × 10^−10^ Bq/L, which corresponds to the lower limit of the range of activity of the clarified phase in storage tanks. In addition, to minimize the volume of the resulting cement compound, it is advisable to consider the issue of pre-drying these LRW approximately two-fold prior to solidification. In this case, the reduction of the Cs-137 activity in the LRW becomes a necessary stage of the technological process for solidification. Moreover, the removal of Cs-137 will significantly reduce the magnitude of gamma fields, which would simplify the process of cementing and subsequent handling of the cement compound.

Sorbents based on ferrocyanides of transition metals extensively used for the removal of cesium from various liquid media [9,10,11,12] are ineffective in processing the clarified part of heterogeneous LRW due to rapid dissolution, which is enhanced in the presence of organic complexing agents [13]. Weakly acidic resorcinol–formaldehyde resins (RFR) are used to remove cesium from alkaline media of various chemical compositions [4,5,14].

The use of RFR with this method is limited by a gradual decrease in sorption-selective characteristics with repeated use in sorption–desorption cycles [4,15]. This is determined by the fact that RFRs tend to be destroyed during prolonged exposure to alkaline media [16,17] with subsequent dissolution due to depolymerization.

It is possible to obtain new properties for the RFR by changing the initial components while preparing the resorcinol–formaldehyde (RF) polymer gel. Taguchi et al. [18] synthesized the resorcinol–formaldehyde (RF) hydrogel with a different ratio between resorcinol and sodium carbonate (R/C) serving as a catalyst for the polymerization reaction. The authors concluded that along with the increase in the R/C ratio, the hydrogel absorption capacity decreases, and the rate of the ion exchange process, on the contrary, increases. Consequently, by changing the resorcinol/formaldehyde ratio at the stage of gel synthesis, it could be possible to obtain the RFRS with increased chemical stability in alkaline media by increasing the degree of crosslinking of a polymer lattice. An important step in the synthesis of RF polymers consists in the subsequent solidification of the gel by heat treatment at a given temperature. Earlier, we showed that the rate of RFR destruction can be decreased by increasing the solidification temperature [19]. Therefore, the objective of the present work was to evaluate the resorcinol/formaldehyde molar ratio during the synthesis of the RF gel and the effect of it and the solidification temperature on the chemical stability and sorption-selective characteristics of the RFR.

## 2. Results and Discussion

### 2.1. Effect of the Resorcinol/Formaldehyde Molar Ratio and the Solidification Temperature on Chemical Stability and Sorption Characteristics of the Resulting Resins

**Figure 1:**
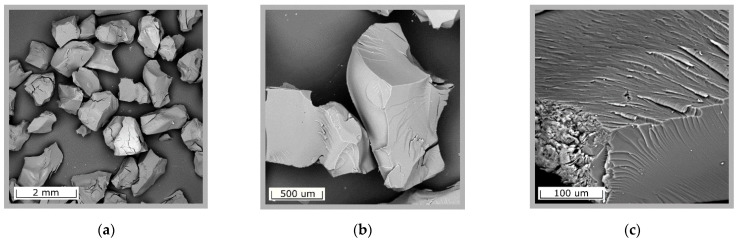
Scanning electron microscopy images of the resorcinol–formaldehyde resins. (**a**) 72× magnification, (**b**) 243× magnification, (**c**) 1500× magnification.

The results of microscopic studies of the RFR 3-1 sorbent are presented in Figure 1. The ion-exchanger surface is monolithic and does not contain cavities or channels. In the process of grinding the solid ion-exchanger, it cracks into irregularly shaped fragments with characteristic faces. The images obtained for RFR 1-1 and RFR 2-1 are the same as for RFR 3-1 and, therefore, are not shown here.

**Figure 2:**
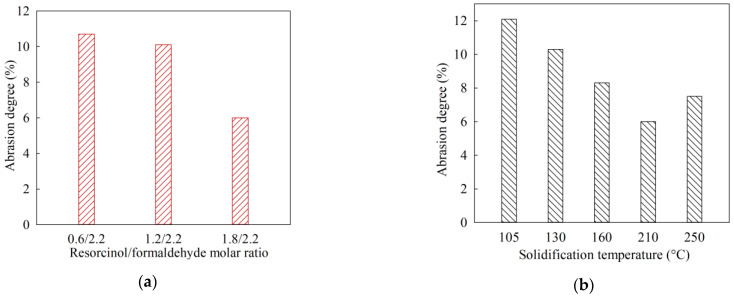
Mechanical strength of dry resorcinol-formaldehyde resins: (**a**) RFR abrasion strength versus resorcinol/formaldehyde molar ratio, (**b**) RFR abrasion strength versus solidification temperature (RFR sample 3-1).

Figure 2 shows diagrams of changes in the RFR mechanical strength depending on the solidification temperature and the molar ratio of resorcinol/formaldehyde. With an increase in the proportion of resorcinol added, the mechanical strength RFR increases, which is indicated by a decrease in the abrasion degree value (Figure 2a). Sample RFR 3-1 has the highest mechanical strength, which was also noted by us during grinding at the sample preparation stage. The effect of solidification temperature on mechanical strength was evaluated using sample RFR 3-1 (Figure 2b). According to the figure, the ion-exchangers mechanical strength increases with rising in the solidification temperature up to 210 °C. At a temperature of 250 °C, thermo-oxidative degradation of the polymer already begins, which negatively affects the RFR mechanical strength.

**Figure 3:**
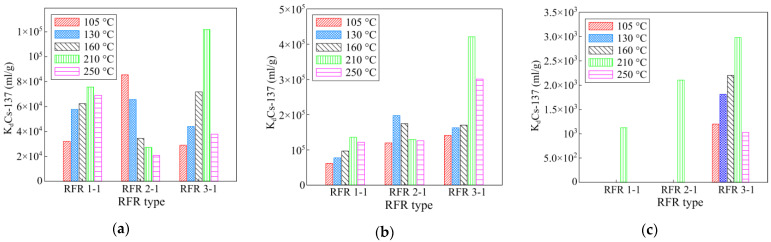
The effect of the solidification temperature and the resorcinol/formaldehyde ratio on the sorption-selective characteristics of the RFR. (**a**) Model solution No. 1, (**b**) model solution No. 2, (**c**) model solution No. 3. The exposure time is 48 h.

A series of the RFR samples with a different resorcinol/formaldehyde ratio and solidification temperature were tested under static conditions in model solutions of various compositions. The experimental results are shown in Figure 3.

The presented diagrams indicate a direct relationship between the sorption-selective characteristics of the RFR and the synthesis conditions. For the RFR 1-1 and RFR 3-1 samples, along with the increase in the solidification temperature, the sorption-selective characteristics increase, which is associated with the course of the solidification process and the further formation of the polymer lattice. The maximum values of the distribution coefficient of RFR 1-1 and RFR 3-1 were observed for the samples solidified at 210 °C. Above 210 °C, the polymer undergoes thermal oxidative degradation, which results in the decrease in the efficiency of radionuclide removal [19]. For RFR 2-1, the dependence of sorption-selective characteristics on the solidification temperature is of a different nature, which is probably related to the peculiar features of the formation of the polymer lattice. In highly alkaline solutions, the model solutions No. 2 and 3 demonstrate the tendency to increase the distribution coefficient along with the increase in the resorcinol/formaldehyde ratio. This effect is most clearly visible in the presence of potassium ions having a greater negative impact on the efficiency of cesium extraction, decreasing the value of the distribution coefficient by one or two orders of magnitude in comparison with sodium ions. Based on the results obtained, further work was carried out using the RFR samples cured at a temperature of 210 °C.

**Figure 4:**
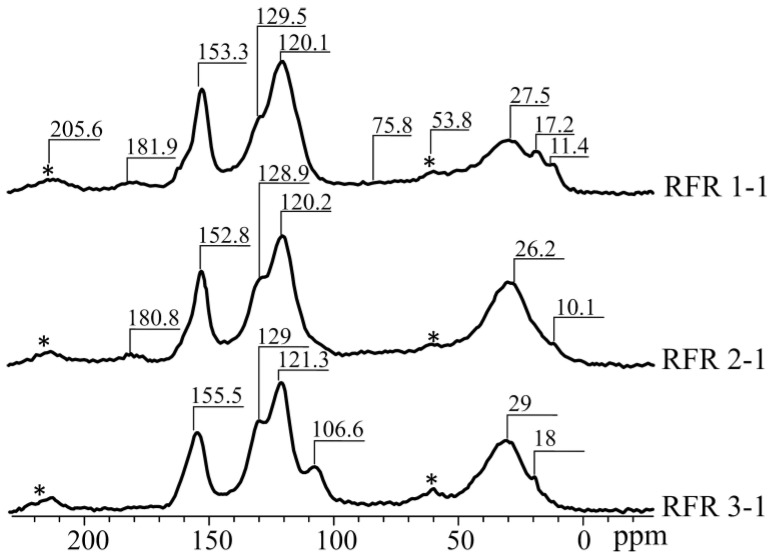
C-13 NMR spectra of RFRs.

Figure 4 shows the C-13 NMR spectra of the RFR samples solidified at 210 °C. The spectra correspond to those described in the published works and contain the following signals: 11.5 CS, 27.5 ppm—methyl and methylene, CS 53.8, 75.5 ppm—oxygen-containing groups (oxydiethylene bridges, etc.), CS 120, 1 ppm—C2, C4, C6, CS 130.8 ppm—S5, CS 138.3 ppm—vinyl, CS 153.5, 155.5 ppm—C1, C3, CS 181.9 ppm—quinones, CS 205.6—aldehydes, ketones [20].

Table 1 shows the values of chemical shifts and the corresponding peak areas. The determination of the relative integral intensities of the spectrum components was carried out by applying an experimental resonance line to a theoretical curve using the least squares method, which was carried out in an independently developed software. The error in determining CS was 0.3 ppm; the intensity of the spectral line was 5% of its area.

Based on the results given in Table 1, it can be concluded that the increase in the resorcinol/formaldehyde molar ratio does not lead to the formation of additional bridge structures connecting resorcinol rings (CS signals at 119, 153, 160), the areas of the peaks corresponding to oxygen-containing groups are of 75.8 and 53.8 ppm (variations within the error). However, it is noteworthy that the samples RFR 1-1 and RFR 2-1 are characterized by the presence of a CS peak at 10 ppm, which probably indicates the break of bridges during polymerization. As for the RFR 3-1 sample, there is no CS peak at 10 ppm, while the CS of 28 ppm is amplified, which indicates the formation of complex bridge structures of the -CH_2_-(CH_2_)_n_ type during the thermal destruction of methylol groups. These bridge structures, probably, contribute to the formation of a strong polymer lattice with a large molecular cell size, which improves the availability of exchange centers.

**Figure 5:**
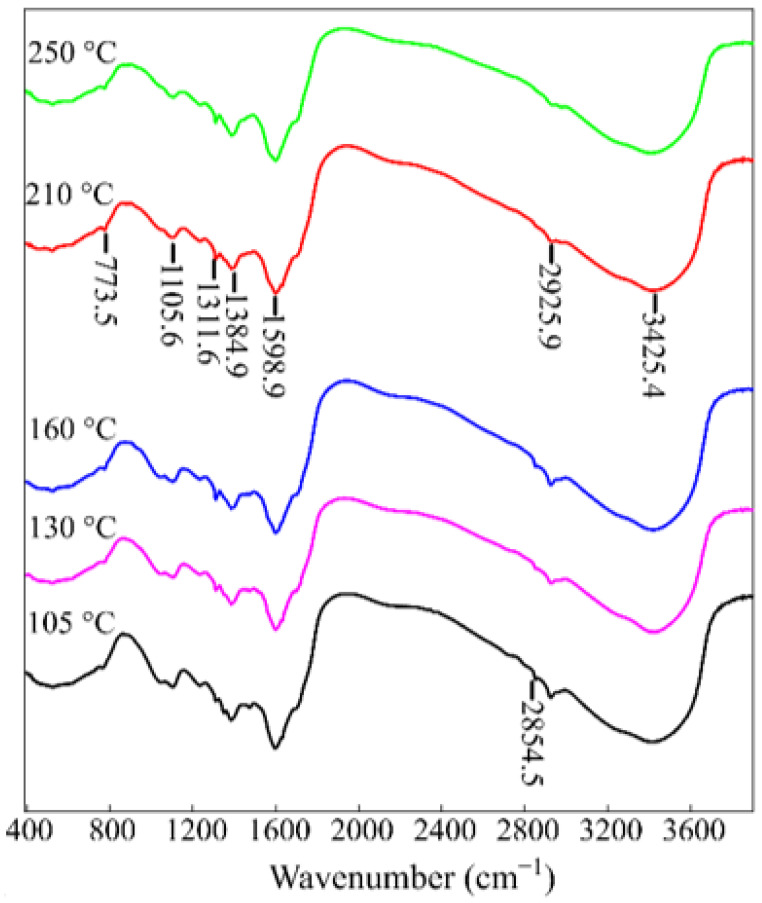
IR spectra of the RFR 3-1 solidified at various temperatures.

Figure 5 shows the IR spectra of the RFR 3-1 samples solidified at various temperatures and containing all the characteristic bands described earlier [21]. In particular, the bands in the range 3700–3300 cm^−1^ correspond to the intra- and intermolecular N-bonds in dimers and polymers. The bands in the range 3200–2800 cm^−1^ correspond to weak (average) stretching vibrations of C-H bonds in the aromatic components, 1608–1400 cm^−1^ correspond to stretching vibrations of the aromatic ring in arenas and acid with H- bonds (formic acid), 1295–1005 cm^−1^ correspond to plane bending vibrations of 1,2-, 1,4-, and 1,2,4-substituted C-H bonds, 1440–1300 cm^−1^ correspond to bending vibrations of OH- groups, 1000–550 cm^−1^ correspond to out-of-plane bending vibrations of C-H bonds in arenes, vibrations of hydroxyl groups, and C-O bonds in carboxylic acids. A distinctive feature for the RFR samples heated at 105, 130, and 150 °C is a band in the vibration range 2400–2000 cm^−1^ corresponding to the bound OH group and stretching vibrations of C-H in carboxylic acids. This fact confirms the presence of a certain number of methylol groups, which indicates incomplete crosslinking of the polymer. The oxidation of such methylol groups to carboxyl groups can occur with the rupture of bridging –CH_2_-groups, which also leads to the destruction of the polymer [22].

**Figure 6:**
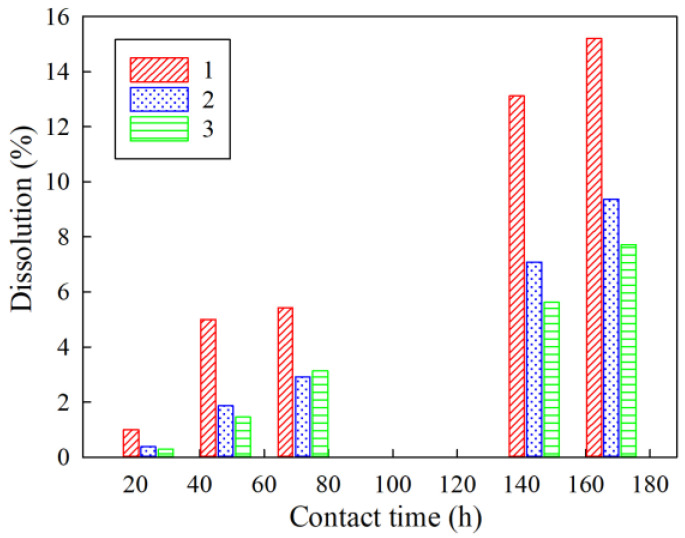
Dependence of the dissolution rate of the RFR samples on the time of contact with the model solution No. 2 (see Table 5). 1– RFR 1-1, 2–RFR 2-1, 3–RFR 3-1.

The increase in the resorcinol/formaldehyde molar ratio contributes to chemical stability to alkaline media, as evidenced by the data obtained during the study of the kinetics of dissolution of sorption materials in contact with a model solution No. 2 under static conditions. Figure 6 shows dissolution diagrams of the RFR, which demonstrate a two-fold increase in the dissolution rate of RFR 1-1 compared to RFR 3-1.

**Figure 7:**
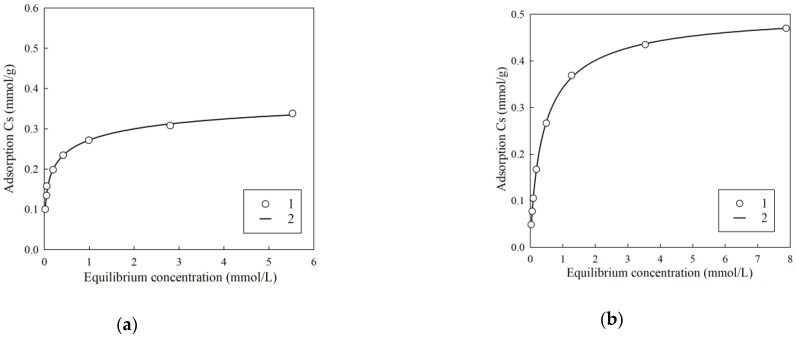
Isotherms of cesium sorption using RFR 3-1. (**a**) Model solution No. 1, (**b**) model solution No. 2. 1—experimental values; 2—approximation of experimental values by the SIPS equation.

Since the total content of Cs (stable isotope Cs-133) in the clarified part of heterogeneous LRW can reach several tens of milligrams per liter [4], an important task is to determine the value of the maximum adsorption, which is calculated using the known equations. Figure 7 shows the isotherms of sorption of stable Cs obtained using model solution No. 1 and No. 2.

Table 2 shows the corresponding values of the parameters of the Sips equation. Taking into account the fact that the experimental values are well described by the Sips equation (*R*^2^ > 0.99), it can be concluded that the heterogeneity of exchange centers has a significant effect on the monolayer filling process. Along with the increase in pH, the increase in the theoretical value of the limiting sorption is observed, which is associated with the deprotonation of functional groups; however, the value of *K_SIPS_* has similar values for both model solutions.

**Figure 8:**
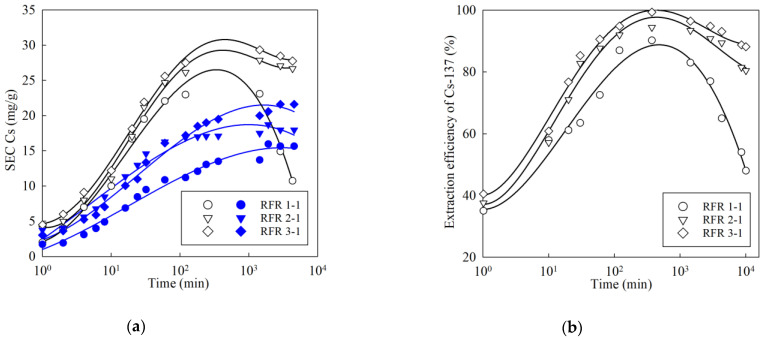
Kinetic curves of cesium adsorption: (**a**) extraction of stable isotope Cs (initial concentration 50 mg/L), (**b**) extraction of micro-concentration of radionuclide Cs-137, initial activity 1000 Bq/mL, open symbols—solution composition: NaNO_3_-2.25 mol/L, NaOH-0.75 mol/L; filled symbols— solution composition: NaOH–10^–4^ mol/L (pH 9).

Figure 8 shows the kinetic sorption curves of the macro-concentrations of the stable isotope Cs-133 (Figure 8a) and the micro-concentrations of the radionuclide Cs-137 (Figure 8b) from various solutions. A common feature here is the increase in the SEC value and the effectiveness of the Cs-137 extraction during the transition from pH 9 to pH 13, which is associated with the increased dissociation of the functional –OH groups of resorcinol. However, in a model solution with pH 13, the curves pass through a maximum in the case of extraction of both the stable isotope Cs and the radionuclide Cs-137. The presence of the maximum is explained by the gradual destruction of the polymer. This process is accompanied by the model solution turning yellow.

Along with the increase in the resorcinol/formaldehyde ratio, the stability of ion-exchangers in solutions with a pH ≥ 13 increases; in addition, the SEC value and the efficiency of Cs-137 extraction increases. It is worth mentioning that in comparison with the RFR 2-1 and RFR 3-1, the kinetic curves for the RFR 1-1 in a solution with pH 13 are characterized by a sharp decrease after reaching a maximum, which indicates the low chemical stability of the ion-exchanger.

**Figure 9:**
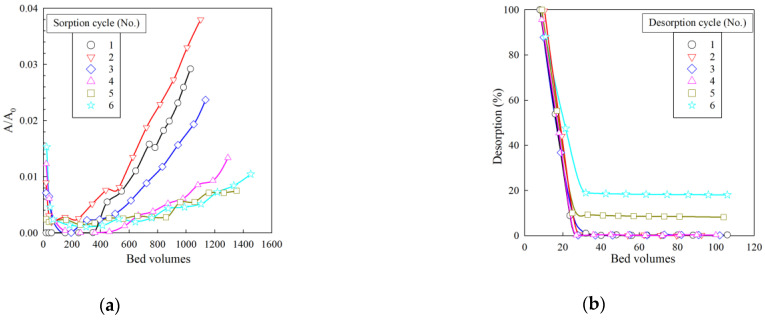
Removal of Cs-137 from model solution No. 2 under dynamic conditions. (**a**) Sorption output curves, (**b**) desorption output curves. *A*_0_—initial solution activity (Bq/mL), *A*—filtrate activity (Bq/mL). Bed volume is the ratio of the resin volume in the column to the fed solution volume.

Figure 9 shows the sorption and desorption curves of Cs-137 micro-concentrations from the model solution No. 2 under dynamic conditions. Along with the increase in the number of sorption cycles, the efficiency of Cs-137 extraction from the model solution gradually increases, which is probably related to the gradual release of the ion-exchanger to the operating mode. The value of the sorption of radionuclide from the model solution in all six sorption cycles exceeds 98%, indicating high efficiency and chemical stability of the RFR 3-1 ion-exchanger.

The desorption curves shown in Figure 9b indicate that, at feeding 35 mL of 1 M HNO_3_ solution, the desorption efficiency exceeds 95% from one to four desorption cycles. In cycles 5 and 6, there is a gradual accumulation of Cs-137 in the resin matrix, which, however, accounts for 3.3% of the total sorbed activity. Based on the data presented, the volume of 1 M HNO_3_ solution can be reduced to 40 mL that enables one to reduce the formation of secondary waste.

### 2.2. Removal of Cesium from a Model Solution Simulating the Clarified Part of Heterogeneous LRW

Table 3 shows the results of the removal and desorption of cesium and potassium under static conditions. Among the components of the clarified heterogeneous LRW, potassium ions are the closest chemical analogs of cesium ions and can have a competing effect on the sorption of the target component, thus occupying the exchange capacity of the resin, which is confirmed by the results shown in Figure 3. For this reason, it is important to understand the efficiency of extraction and desorption of potassium, which can accumulate in the resin volume.

According to the presented results, the SEC value for potassium is higher than for cesium. These results can be explained by the difference in the initial concentrations of potassium and cesium in the model solution. The excess potassium content is compensated by the selectivity of RFR to cesium, as indicated by the values of the distribution coefficient. Despite the fact that the affinity of RFR is more pronounced to cesium, an unexpected result is the fact that potassium is practically not desorbed from the RFR under experimental conditions. The maximum value of the degree of desorption was only 3.3% (Table 3).

**Figure 10:**
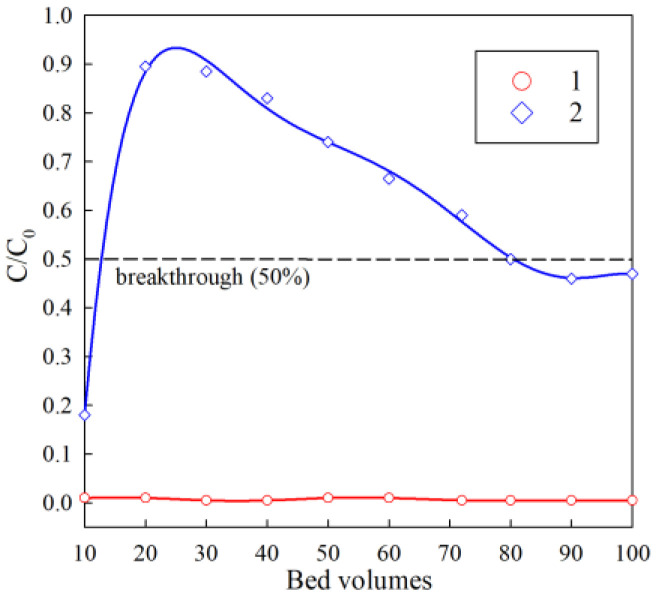
Output curves of the sorption of cesium and potassium in the experimental dilute solution. 1—cesium extraction curve; 2—potassium extraction curve; *C*_0_ is the initial concentration (mg/L), *C* is the concentration in the filtrate. Bed volume is the ratio of the resin volume in the column to the fed solution volume.

The results of the study of the removal of cesium from a dilute model solution simulating the clarified part of heterogeneous LRW are shown in Figure 10. At the sorption stage, 100 bed volumes of the model solution were passed through the column with resin. High coefficients of solution purification from cesium were preserved consistently throughout the sorption cycle. The efficiency of cesium extraction was also at a high level and amounted to more than 99%. Saturation of the sorbent with cesium was not achieved during the experiment. There are some features in the potassium extraction under dynamic conditions. A sharp rise in the initial part of the adsorption curve can be explained by the fact that an excess of potassium ions leads to the rapid filling of exchange centers, which are located at the RFR grain surface. The adsorption centers filling is accompanied by a rapid decrease in the potassium extraction efficiency already after 20 bed volumes. A further gradual increase in the potassium extraction efficiency can be associated with the reaching of RFR to the operating mode due to gradual swelling, which simplifies the intra-particle diffusion of potassium. The desorption degree for cesium exceeded 99%; for potassium, it was less than 1%. In theory, despite the high selectivity and chemical stability, the accumulation of potassium in the ion-exchanger grain volume can lead to the decrease in the ion-exchanger filter cycle resource. This phenomenon requires further research.

## 3. Conclusions

The chemical stability and sorption characteristics toward Cs-137 depend on the resorcinol/formaldehyde molar ratio, as well as the solidification temperature of the RF gel. Along with the increase in the resorcinol/formaldehyde molar ratio (0.6/2.2–1.2/2.2–1.8/2.2), the chemical stability of the RFR improves, which has a positive effect on its sorption characteristics in liquid alkaline media. The increase in the solidification temperature results in the increase in the chemical stability (with the exception of the RFR 2-1), as well as sorption characteristics. However, the solidification temperature must not exceed 210 °C, which is when the thermal oxidative degradation of the polymer begins. It has been demonstrated that the increase in the chemical stability of the RFR samples occurs due to the increase in the degree of crosslinking of the polymer lattice.

Under dynamic conditions, in six sorption–desorption cycles, the efficiency of Cs-137 extraction from the decontamination solution exceeded 98%, while there was no noticeable destruction of the ion-exchanger. Prolonged use of the ion-exchanger leads to the accumulation of the non-resorbable part of Cs-137, which, however, does not exceed 3.3% of the total sorption activity. Accumulation can be associated with low diffusion coefficients.

Experiments with model solutions simulating the clarified part of heterogeneous LRW have shown the high efficiency and prospects of the sample RFR 3-1. However, the adsorption of a certain amount of potassium was detected, which is practically not desorbed by a solution of HNO_3_.

## 4. Materials and Methods

### 4.1. RFR Synthesis

The RFR samples were synthesized according to the following scheme. A given weight sample of resorcinol and 2.8 g of KOH were dissolved in distilled water and cooled down to 5–7 °C. Formalin was added to the resulting solution under intensive stirring for 2 min. As a result, the reaction mixture became brown, which indicates the formation of an oligomeric mixture. During 10–15 min, the process of polymerization of the oligomeric mixture occurred with the formation of the resorcinol–formaldehyde polymer gel of brown color with a reddish tint. The resulting gels were heated at preset temperatures in air for 6 h. During the heating process, the polymer solidified, which was accompanied by further crosslinking of the polymer. As a result, gel samples with different resorcinol/formaldehyde molar ratios were prepared. It is worth mentioning that the gel formation does not occur at the following molar ratios: 0.1/2.2 and 0.3/2.2, whereas the reaction mixture remains liquid for a long time. The synthesis conditions and denotation of the RFR samples are given in Table 4.

The solidified ion-exchangers were further mechanically ground, after which they were transferred into the H-form by sequential washing with the following solutions in the following under dynamic conditions: HNO_3_ (1 mol/L), NaOH (0.5 mol/L), and HNO_3_ (1 mol/L).

### 4.2. Experimental Technique

#### 4.2.1. Evaluation of the Effectiveness and Chemical Stability of RFR

The experiments were carried out using both a micro-concentration of Cs-137 and macro-concentration of the stable isotope Cs-133. Before the experiment, the resins were held for several hours in model solutions without cesium in order to achieve chemical equilibrium. V/m = 1000 mL/g (V is the volume of the liquid phase, m is the mass of ion-exchanger), grain size 0.1–0.2 mm. Table 5 shows the compositions of model solutions that were used in the work.

After exposure, the RFR samples were placed into model solutions with a preset concentration of the stable cesium isotope or the activity of Cs-137. At specified intervals, the solution was separated from the RFR by decantation, and the residual concentration and activity were determined.

The removal efficiency was calculated according to Equation (1):

(1) $$S=\left(1-\left(\frac{A_{e}}{A_{i}}\right)\right)\times 100.$$

The value of the Cs-137 distribution coefficient (mL/g) was calculated using Equation (2):

(2) $$K_{d}=\frac{A_{i}-A_{e}}{A_{i}}\times \frac{V}{m}$$

where *A_i_* is the initial activity of Cs-137 in the solution (Bq/L), *A_e_* is the equilibrium residual activity of Cs-137 in the solution (Bq/L), *V* is the volume of the solution (mL), and *m* is the weight of the sorbent sample (g).

The static exchange capacity (SEC, mg/g) was calculated according to the following Equation (3):

(3) $$SEC=\frac{\left(C_{i}-C_{e}\right)\times V}{m}$$

where *V* is the volume of the solution (L), *C_i_* is the initial concentration of cesium (mg/L), *C_e_* is the equilibrium concentration of cesium (mg/L), and *m* is the sorbent mass.

The value of the limiting capacity was estimated by the sorption isotherms, which were obtained by bringing samples into contact with a series of model solutions containing a given amount of stable isotope Cs. The duration of mixing is 48 h.

The Sips Equation (4) was used to describe the sorption isotherms:

(4) $$\Gamma =G_{max}\times \frac{K_{l}\times C^{m}}{1+K_{l}\times C^{m}}$$

where *G_max_* is the maximum sorption value (mmol/g), *C* is the equilibrium concentration of Cs (mmol/L), *K_SIPS_* is the adsorption equilibrium constant characterizing the energy of the adsorbent–adsorbate bond, and *m* is an indicator of the heterogeneity of exchange centers characterizing the change in the heat of adsorption depending on the degree of their filling.

The kinetic characteristics of the ion exchange process were evaluated using model solutions containing preset concentrations of stable cesium (50 mg/L) or activity of Cs-137 (500–1000 Bq/mL).

The chemical stability of ion-exchangers in alkaline solutions was evaluated by analyzing the organic solution. The content of organic carbon in solutions was determined by the modified Tyurin method [24]. First, 5 mL of 0.4 N solution of potassium bichromate in sulfuric acid (1:1) was added to 1 mL of the test solution and held at 150 °C for 20 min. After cooling, the sample volumes were adjusted to 10 mL, and the optical density was measured in the obtained solutions at a wavelength of 590 nm relatively to the background solution using a Shimadzu 1800 PC spectrophotometer (Shimadzu, Japan). The calibration curve for determining the carbon content of 0.015–2 g/L was obtained using the resorcinol solutions, assuming that 1 g of resorcinol contains 0.65 g of organic carbon.

The mass fraction of organic matter (*R*) as a percentage was calculated by Equation (5):

(5) $$R=\frac{C_{0}-C}{C_{0}}\times 100\%$$

where *C*_0_ and *C* are the concentrations of organic carbon (g/L) in the model solution before and after the introduction of the RF resins, respectively, which was determined by the Tyurin method.

The efficiency of cesium removal and desorption, as well as the stability of the RFR samples, were evaluated under dynamic conditions in repeated sorption–desorption cycles. The volume of the ion-exchanger in the column was 1 mL, the inner diameter of the column was 4 mm, and the percolation rate was 25 BV/h (bed volumes per hour). Desorption of the accumulated radionuclide with RFR was carried out with a solution of HNO_3_ of a concentration of 1 mol/L at a percolation rate of 12.5 BV/h. The desorption efficiency was calculated by Equation (6).

(6) $$\%Desorption=\frac{\sum_{1}^{i}(V_{ki}\times A_{ki})}{A_{c}}\times 100$$

where *A_c_* is the RFR activity (Bq), *A_k_* is the eluate activity (Bq/mL), *V_k_* is the volume of eluate (mL), and *i* is the ordinal number of the eluate fraction.

#### 4.2.2. Removal of Cesium from a Model Solution Simulating the Clarified Part of Heterogeneous LRW

An experimental solution simulated the chemical composition of the alkaline decantate of the accumulated highly-active waste. The preparation of the model solution was carried out in accordance with the procedure described by Glagolenko et al. [25]. The cationic composition of the model solution is presented in Table 6.

The RFR weight samples were brought into contact with the model solution under continuous stirring for 6 h, the V/m ratio was 100 mL/g, while the weight of the resin sample was 0.1 g. After a specified time, the residual contents of cesium and potassium were determined in the solution. Desorption was carried out with HNO_3_ solution of a concentration of 0.5 mol/L, the V/m ratio was 100 mL/g, and the exposure duration was 6 h. After a specified time, the concentrations of cesium and potassium were determined in the solution.

The efficiency of cesium removal under dynamic conditions was evaluated as follows. A chromatographic column (100 × 4.6 mm) was filled with the prepared resin. To do this, the resin was placed in a column in small portions using a plastic funnel and compacted by intensive shaking. Prior to the experiment, the model solution was diluted 4-fold in order to minimize the negative effect on the resin of the alkaline solution.

The rate of the process at the sorption stage was 3 BV (bed volumes)/h; at the desorption stage, it was 3 BV/hour. For desorption, 0.5 mol/L HNO_3_ solution was used. The concentrations of cesium and potassium were determined in the filtrate and eluate at the outlet of the column.

The mechanical strength was evaluated using a Pulverisette 0 vibration mill (Fritsch, Germany) with a bowl and a grinding ball of agate. For this, a weighed portion of a dry sample with a grain size of 0.1–0.2 mm was placed in a bowl and milled for 20 min. The experiment was repeated three times for each RFR sample in order to evaluate the results’ repeatability. After milling, re-sieving was carried out, and the resin mass with a grain size of less than 0.1 mm was determined. The abrasion degree RFR was calculated using Equation (7):

(7) $$Abrasion\;degree=\left(\frac{m_{a}}{m_{t}}\right)\times 100$$

where *m_a_* is the mass of resin with a particle size less than 0.1 mm (g), and *m_t_* is the initial mass of the sample (g).

### 4.3. Equipment and Software

The Cs-137 activity in the solution was determined by a direct radiometric method using an RCG-AT1320 gamma radiometer with an NaI(Tl) 63 × 63 mm detector (NPP Atomtech, Republic of Belarus, Minsk).

Microscopic studies of ion-exchangers were carried out using a Mira3 LMH scanning electron microscope by TESCAN.

The contents of elements in the model solution were evaluated by the atomic absorption flame spectroscopy using a Thermo Solar AA M6 instrument (Thermo Electron Corporation, Waltham, WA, USA). The surface morphology of the samples was studied by scanning electron microscopy (SEM) using a Carl Zeiss Crossbeam 1540-XB device (Oberkochen, Germany).

The calculations were performed using SciDAVis 1.23 (for Windows), the release date 4 June 2018, GNU General Public License.

The ^13^C NMR spectra of the polymers in the solid phase were recorded using a Bruker Avance AV-300 device (Billerica, MA, USA) with a proton resonance frequency of 300 MHz. To record the spectra, the method of rotating samples at a magic angle (VMU) was used. Tetramethylsilane was used as a standard for carbon nuclei; the chemical shift zero (CS) was set in a separate experiment. The spectra were recorded at 300 K.

## Figures and Tables

**Table 1:** Values of chemical shifts and corresponding peak areas on the C-13 NMR spectra of the RFR samples heated at 210 °C.

Peak No.	Organic Group	RFR 1-1		RFR 2-1		RFR 3-1	
		CS, ppm	Area (%)	CS, ppm	Area (%)	CS, ppm	Area (%)
1	–CH_3_	9.85	3.50	10.05	2.85	–	–
2	>HC-CH_3_	15.71	2.66	26.23	38.77	17.33	1.23
3	–CH_2_–CH_2_-	27.24	27.64	–		28.45	34.75
4	–CH_2_(OCH_2_)_x_OH	55.25	3.32	49.79	3.23	48.48	3.25
5	–CH_2_–O–CH_2_–	78.39	1.35	75.77	1.26	75.67	1.22
6	˃HC–H	–	–	–	–	106.29	4.03
4	C2, C4, C6	119.02	36.17	119.83	28.71	120.24	25.05
8	C1, C3	152.08	15.02	153.09	12.59	154.91	14.94
9	C1, C3	160.67	3.04	160.37	3.00	161.88	2.48
10	–CH=O	180.08	1.05	180.99	1.02	180.92	0.98

**Table 2:** Calculated parameter values for the Sips equation.

Parameter	Model Solution	
	No. 1	No. 2
*G_max_*	0.42 ± 0.04	0.51 ± 0.01
*K_SIPS_*	1.8 ± 0.5	2.1 ± 0.1
*m*	0.43 ± 0.05	0.85 ± 0.02
*R* ^2^	0.994	0.999

**Table 3:** Results of sorption extraction and desorption of cesium and potassium on the resorcinol-formaldehyde resin under static conditions, V/m-1000 mL/g, contact time 6 h.

Cesium			Potassium		
*K_d_* (mL/g)	SEC (mmol/g)	Desorption Efficiency (%)	*K_d_* (mL/g)	SEC (mmol/g)	Desorption Efficiency (%)
187	0.03	75	110	1.07	3.3

**Table 4:** Resorcinol-formaldehyde resins synthesis and denotation of the samples.

Denotation	RFR 1-1 ^1^	RFR 2-1	RFR 3-1
Volume ratio of resorcinol/formalin solution	1/1	2/1	3/1
Resorcinol/formaldehyde molar ratio	0.6/2.2	1.2/2.2	1.8/2.2

^1^ Bibler et al. [23].

**Table 5:** Compositions of the model solutions.

Model Solution (No.)	Composition (mol/L)
1	NaOH–10^−4^
2	NaOH–0.75, NaNO_3_–2.25
3	KOH–0.75, KNO_3_–2.25

**Table 6:** Cationic composition of the model solution [12].

Element	Content (g/L)
Na	94
Al	5.6
K	0.7
Cs	0.055
NO_3_	102
NO_2_	33.4
SO_4_	14.1
CrO_4_	1.1
SiO_3_	0.55

## Data Availability

Not applicable.
